# *l*-Borneol Exerted the Neuroprotective Effect by Promoting Angiogenesis Coupled With Neurogenesis via Ang1-VEGF-BDNF Pathway

**DOI:** 10.3389/fphar.2021.641894

**Published:** 2021-03-05

**Authors:** Rong Ma, Qian Xie, Hongyan Li, Xiaoqing Guo, Jian Wang, Yong Li, Mihong Ren, Daoyin Gong, Tian Gao

**Affiliations:** ^1^ State Key Laboratory of Southwestern Chinese Medicine Resources, Chengdu, China; ^2^ School of Pharmacy, Chengdu University of Traditional Chinese Medicine, Chengdu, China; ^3^ Department of Pathology, Hospital of Chengdu University of Traditional Chinese Medicine, Chengdu, China; ^4^ Adverse Reaction Monitoring Center, Hospital of Chengdu University of Traditional Chinese Medicine, Chengdu, China

**Keywords:** l-borneol, cerebral ischemia, pMCAO, angiogenesis, neurogenesis

## Abstract

At present, Stroke is still one of the leading causes of population death worldwide and leads to disability. Traditional Chinese medicine plays an important role in the prevention or treatment of stroke. *l*-borneol, a traditional Chinese medicine, has been used in China to treat stroke for thousands of years. However, its mechanism of action is unclear. After cerebral ischemia, promoting angiogenesis after cerebral ischemia and providing nutrition for the infarct area is an important strategy to improve the damage in the ischemic area, but it is also essential to promote neurogenesis and replenish new neurons. Here, our research shows that *l*-borneol can significantly improve the neurological deficits of pMCAO model rats, reduce cerebral infarction, and improve the pathological damage of cerebral ischemia. and significantly increase serum level of Ang-1 and VEGF, and significantly decrease level of ACE and Tie2 to promote angiogenesis. PCR and WB showed the same results. Immunohistochemistry also showed that *l-*borneol can increase the number of CD34 positive cells, further verifying that *l-*borneol can play a neuroprotective effect by promoting angiogenesis after cerebral ischemia injury. In addition, *l-*borneol can significantly promote the expression level of VEGF, BDNF and inhibit the expression levels of TGF-β1 and MMP9 to promote neurogenesis. The above suggests that *l-*borneol can promote angiogenesis coupled neurogenesis by regulating Ang1-VEGF-BDNF to play a neuroprotective effect. Molecular docking also shows that *l-*borneol has a very high binding rate with the above target, which further confirmed the target of *l-*borneol to improve cerebral ischemic injury. These results provide strong evidence for the treatment of cerebral ischemia with *l-*borneol and provide reference for future research.

## Introduction

Stroke which caused by interruption to flow of blood in brain vessels results in the insufficient blood supplement. Stroke is still one of the leading causes of population death worldwide and leads to disability at present (Phipps and Cronin, 2020). It has the characteristics of high morbidity, high lethality rate, and high disability rate, and there is a tendency of rejuvenation (Boot et al., 2020). The outcomes of ischemic stroke damage are far-reaching, generating immense burden to both the family and society (Roth et al., 2018). So far, ideal drugs or strategies for ischemic stroke are still unavailable. Present strategies for treatment of stroke include recanalization by means of pharmacologic or mechanical thrombolysis and neuroprotective agents (Alim et al., 2019). Tissue plasminogen activator (t-PA) intravenous thrombolysis has been approved as the principal recommended strategy for the treatment of acute stroke by US Food and Drug Administration (FDA) (Dirnagl et al., 1999; Donnan et al., 2008) The reports stated intravenous thrombolysis within 6 h after the onset of stroke is the only treatment to reduce the disability of stroke patients (Miller et al., 2012; Wardlaw et al., 2012; Demaerschalk et al., 2016; Furie and Jayaraman 2018). However, its activation of the fibrinolytic system caused bleeding and limited its application (Külkens and Hacke, 2014; Hendersonet al., 2018). According to reports, only 9.9% of patients can receive thrombolytic therapy, and there are some patients with unsuccessful thrombolysis. Hence, there is necessary to explore novel neuroprotective strategies for ischemic stroke treatment. Further research is needed to identify neuroprotective drugs and their mechanisms, which will prevent or ameliorate brain injury.

Emerging evidence suggests the cerebral ischemia injury caused a sophisticated cascade of pathophysiologic events (Kahles and Brandes 2013; Ludewig et al., 2019). After cerebral ischemia, brain parenchyma cells, intercellular stroma and microvascular regeneration is inhibited, which leads to the edema and death of glial cells and vascular cells in the acute phase, and even causes the injured neurons to lose timely nutritional support and die. Therefore, vascular reconstruction is very important to save reversible nerve injury of ischemic penumbra and promote regeneration and repair after injury (Wang et al., 2019). Recent studies have shown that angiogenesis plays an important role in neuroprotection (Seevinck et al., 2010; Benedek et al., 2019). Ischemia can cause neuronal ischemia and hypoxia death, so it is also essential to promote neurogenesis and replenish damaged neurons (Zhang et al., 2020). The ideal strategies are to promote angiogenesis, improve microcirculation, increase cerebral blood flow, and promote neurogenesis, replenish damaged neurons, repair brain tissue injury in ischemic area, further to improve neurological dysfunction (Lunardi and Lehmann 2019). In fact, angiogenesis and neurogenesis often go hand in hand. The new neurons can guide the development of the vascular tree to the infarct area and improve the tissue perfusion in the infarct area and penumbra. The new blood vessels can provide nutrients for the new neurons, promote their maturation, and promote their migration to the infarct area.

Vascular endothelial growth factor (VEGF) is considered to be the most powerful and specific angiogenic factor, while angiopoietin (Ang) is the first one that has been determined to have the effect of promoting angiogenesis. VEGF and Ang coordinate with each other and jointly regulate angiogenesis (Zhang and Chopp 2002). Another study showed that VEGF-Ang/Tie pathway has important regulatory significance in cerebral ischemia (Hori et al., 2004). Brain derived neurotrophic factor (BDNF) is a kind of neurotrophic factor formed in the brain, which is involved in the survival, differentiation, and maturation of neurons (Liu et al., 2020; Müller et al., 2020). What’s more, BDNF is the possible mechanism by exercise exerts positive effects in the brain (Amin et al., 2020). VEGF also has the effect of promoting neurogenesis, especially in the hippocampus and subventricular zone (SVZ) area (Greenberg and Jin, 2013). VEGF can stimulate axon growth, promote the proliferation and differentiation of neuron precursors, and promote the release of BDNF from endothelial cells (Sun et al., 2003). Simultaneously, VEGF-BDNF pathway could improve the cognitive dysfunction (Jeong et al., 2019). TGF-β1 also played an important role for ischemic cerebrovascular disorders (Rehab et al., 2020). Therefore, angiogenesis and neurogenesis might be a potential mechanism to improve cerebral ischemia injury.

**FIGURE 1:**
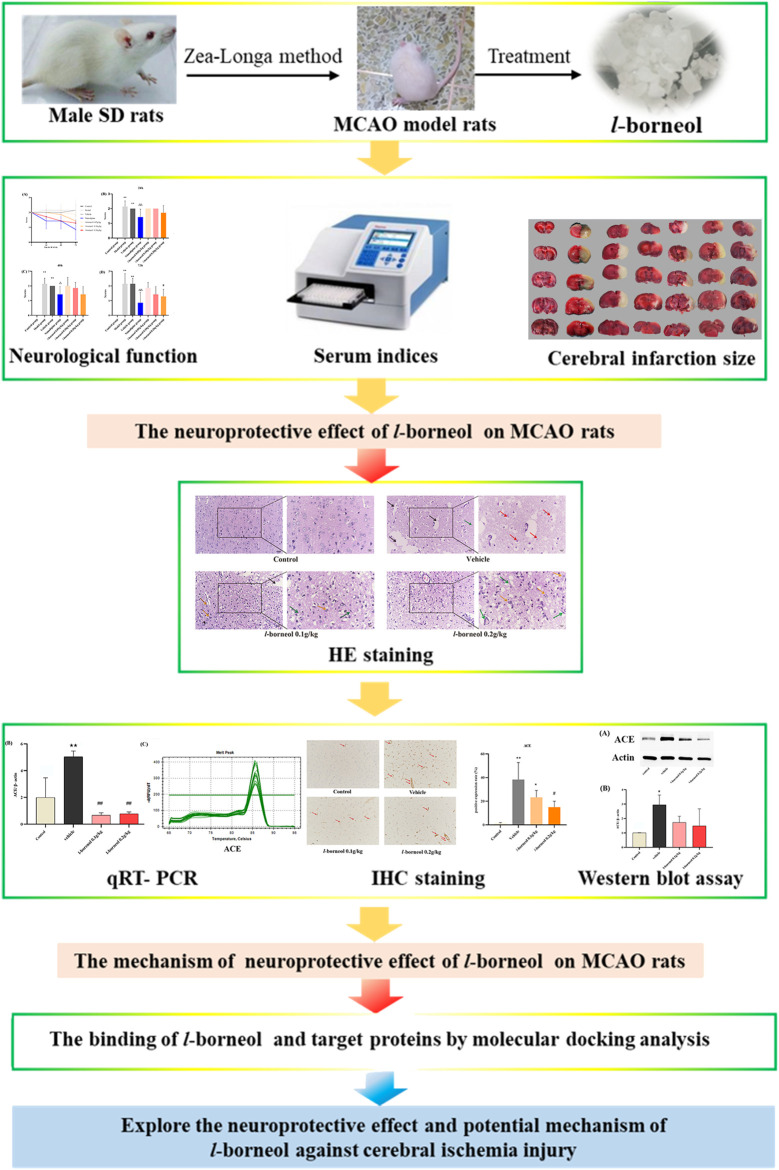
The graphical abstract of this study.

Borneol is a traditional Chinese medicine, which has been used to treat stroke for a long history in China. In recent years, it was reported that borneol has many pharmacological effects, such as, anti-inflammatory, analgesic, sedative, and anti-bacterial, anti-tumor (Sousa et al., 2007; Chen et al., 2019; Cao et al., 2020; Ji et al., 2020; Xin et al., 2020). Our previous studies showed that borneol could reduce the temperature of lipopolysaccharide (LPS) induced fever rats (Luo et al., 2016). Both *l-*borneol and synthetic borneol had a brain protection effect and regulated the permeability of blood brain-barrier (BBB), and the effect of *l-*borneol was better than synthetic borneol (Ni et al., 2011a; Ni et al., 2011b; Tian et al., 2013; Zhou et al., 2014). Simultaneously, we found that borneol could significantly increase the serum VEGF in the middle cerebral artery occlusion (MCAO) rats, and reduce the TNF-α level, and borneol could improve the neuron BBB ultrastructure to protect neurovascular units (Dong et al., 2018). However, the mechanism of *l-*borneol has not been elucidated. Therefore, it is the first time to investigate the mechanism of *l-*borneol by angiogenesis and neurogenesis. This study might provide a novel view on the neuroprotective effect of *l-*borneol on stroke. The graphical abstract of this study is shown in Figure 1.

## Materials and Methods

### Animals

Male Sprague Dawley (SD) rats weighing (8–10 weeks old, 250 ± 10 g) were obtained from SPF (Beijing) Biotechnology Co., Ltd. (Beijing, China), permit number: SCXK (Jing) 2019-0010. All animals were handled in keeping with institutional guidelines and ethics. Rats were housed at 22 ± 2°C with a 12 h light/12 h dark cycle. The animals had free access to food and water. Randomization was used to allocated animals to various experimental groups and the data analysis were performed by a blinded investigator. The experimental processing is approved by ethics committee of Affiliated Hospital of Chengdu University of Traditional Chinese Medicine, number: DL2019002.

### Experimental Drugs and Reagents

The *l*-borneol was purchased from Luodian, Guizhou, China, which is a high-quality production area for *l*-borneol. According to the method specified in the Pharmacopoeia of the People’s Republic of China, 88.6% of the *l*-borneol in this study meets the requirement of no less than 85.0%. Nimodipine was obtained from Yabao Pharmaceutical Group Co., Ltd. (Shanxi, China). Hematoxylin (batch number, G1140) and Eosin (G1100) was purchased from Solarbio co., Ltd. (Beijing, China). 2,3,5-Triphenyltetrazolium chloride (TTC) was purchased from Kelong co. (Chengdu, China). Brain derived neurotrophic factor (BDNF) (Lot No. 9ZFHS54AQT), Tie2 (Lot No. M6RQRBWEEN), Matrix metalloprotein 9 (MMP 9) (Lot No. YU5E7JW4UR) and Ang-1 (Lot No. JNS67D5Z7W) kits were purchased from Elabscience Biotech Co., Ltd. (Wuhan, China). Vascular endothelial growth factor (VEGF) (Lot No. A38381252) and TGF-β1 (Lot No. A18181155) kits were purchased from MultiSciences (Lianke) Biotech Co., Ltd. (Hangzhou, China).

### Drug Preparation

The borneol dosing was calculated according to the dose conversion table of experimental animals. The 10 times of an adult’s (60 kg) daily consumption (0.3 g) is as equivalent dosing (0.05 g/kg). The 1, 2, four time(s) of equivalent dose were used as low dosing (0.05 g/kg), medium dosing (0.1 g/kg) and high dosing (0.2 g/kg). Every group were dissolved in 5% tween 80 solution. The concentration of nimodipine was 0.012 g/kg, which is dissolved in pure water.

### Permanent MCAO Surgery, Neurological Deficit Determination and Drug Treatments

Rats were adaptively fed for 5 days. A total of 54 male rats were divided into two parts of the experiment. The first part included the sham operation group, model group, vehicle group, nimodipine group, and *l-*borneol 0.05 g/kg group, *l-*borneol 0.1 g/kg group, *l-*borneol 0.2 g/kg group, with six rats in each group. The second part includes sham operation group, vehicle group, *l-*borneol 0.1 g/kg group, *l-*borneol 0.2 g/kg group, with three rats in each group. The rats were subjected to permanent MCAO surgery as described in previous study (Dong et al., 2018; Ma et al., 2020). The rat body temperature was kept at 37°C during surgery. The skin incision and blood vessel dissection only were performed in the sham operation (Control) group, and the rest of the operation was the same as the other groups. Perform neurobehavioral testing by the classic neurologic deficit score method Zea-Longa to determine the neurological deficit after MCAO (Table 1). For the first part of the experiment, then grade 2 of MCAO animals were randomly divided into six group: model group, vehicle group, nimodipine group, *l-*borneol 0.05 g/kg group, *l-*borneol 0.1 g/kg group, *l-*borneol 0.2 g/kg group. All of rats were treated by intragastric administration with 10 ml/kg. The control group and model group were given the same volume of pure water. The vehicle group was given the same volume of 5% tween 80 solutions. The nimodipine group, *l-*borneol 0.05, 0.1, 0.2 g/kg group were given the same volume of the corresponding drug solution. All rats were given continuous intervention for 3 days, and neurobehavioral tests were performed at 24, 48, and 72 h after MACO. In the second part of the experiment, rat brain tissue is used for IHC and PCR.

**TABLE 1:** Zea-Longa neurological function score.

Grade	Rat behavior	Neurological function
0	No neurological deficit	Normal
1	The forelimb on the paralyzed side cannot be fully extended	Mild neurological deficit
2	The rat turns to the paralyzed side while walking	Moderate neurological deficit
3	The rat falls to the paralyzed side while walking	Severe neurological deficit
4	Can't go spontaneously, lose consciousness	Very severe neurological deficit

### Serum and Brain Tissue Preparation

After neurological test, the rats were anesthetized and decapitated under anesthesia. Thereafter, the serum was obtained from abdominal aortathe and centrifuged 3,000 rpm after standing. The supernatant was collected and frozen at −80°C. The rat brains were promptly obtained and the olfactory bulb, lower brain stem and cerebellum were removed.

### Cerebral Infarction Assessment

The brain was frozen at −20°C for 15 min. Five coronal brain sections of 2 mm thickness were stained with TTC at 37°C for 30 min in darkness. After TTC staining, those pieces were fixed with 4% formaldehyde for 24 h in the dark. The cerebral infarction rate was analyzed.

### Enzyme-Linked Immunosorbent Assay (ELISA) Detection

The supernatant serum had been obtained. levels of VEGF, Ang1, BDNF, Tie2, TGF-β1, MMP9 were detected according ELISA kits complied with the manufacturer's protocol.

### Hematoxylin-Eosin (HE) Staining

The brain tissue was fixed in 4% paraformaldehyde and embedded in paraffin. The paraffin-embedded brain tissue was cut into slices. The slices were dehydrated and dewaxed and then stained with HE for morphological evaluation. Each slice was observed under ×200 and ×400 microscopy. According to the scoring standards of semi-quantitative injury and repair in Table 2 and Table 3, the pathological conditions of brain tissue in each group were analyzed.

**TABLE 2:** Semi-quantitative injury score standards.

Scores	0	1	2	3
Liquefying necrosis of infarct	−	+	++	+++
Red neuron	−	+	++	+++
Inflammatory cell infiltration	−	+	++	+++

**TABLE 3:** Semi-quantitative repair score standards.

Scores	0	1	2
Vessels number	−	+	++
Phagocyte	−	+	++

According to the degree of pathological changes from light to heavy, the semi-quantitative results are as follows: slight or very small "-" marked 0, slight or small "+" marked 1, moderate "+" marked 2, severe or large "+ +" marked 3.

According to the degree of repair from bad to good, the semi-quantitative results are as followed: slight or very small "-" marked 0, slight or small "+" marked 1, moderate or medium "+ +" marked 2.

### Quantitative Real-Time Polymerase Chain Reaction (qRT-PCR)

Total RNA was obtained with the Trizol Reagent, which was reverse transcribed to cDNA with the Servicebio^®^ RT First Strand cDNA Synthesis Kit (Servicebio. Co., Ltd., G3330, Wuhan, China). The CFX96 real-time PCR detection system (Bio-Rad Laboratories Ltd., Hertfordshire, United Kingdom) was used to perform qRT-PCR. The specific primers and according sequences were displayed in Table 4. In the reaction, 1 μL cDNA of each sample was mixed with Servicebio^®^ 2 × SYBR Green qPCR Master Mix (None ROX) (Servicebio. Co., Ltd., G3320, Wuhan, China) according to the manufacture’s protocol. The PCR conditions were listed as follow: 30 s at 95°C, then 40 cycles at 95°C for 15 s, followed by 60°C for 30 s. Results were normalized to Actin mRNA level and presented as the fold change (2^–ΔΔCt^).

**TABLE 4:** Sequence of primers.

Primer	Sequence (5′->3′)
ACE	F: TTC​ACA​TCC​CAA​GCG​TGA​CA, R: CTG​AAC​CCA​CCA​GGT​CCT​TC
CD 34	F: CAG​TCT​GAG​GTT​AGC​CCG​A, R: CTC​GGG​TCA​CAT​TGG​CCT​TTC
HIF 1α	F: GCG​GCG​AGA​ACG​AGA​AGA​AA, R: AGA​TGG​GAG​CTC​ACG​TTG​TG

### Immunohistochemical (IHC) Staining

IHC was used to determine the expression of anti-angiotensin-converting enzyme (ACE) and CD34 protein. In brief, the brain sections with paraffin-embedded (4 μm) were rinsed with 3% H_2_O_2_ for 10 min to block endogenous peroxide activity and incubated with 10% goat serum albumin for 30 min to block nonspecific binding. Then, the sections were incubated with the primary antibodies (ACE, abcam, No. ab254222, United Kingdom, 1:100) and anti-CD34 (1:200) overnight at 4°C, after incubation, sections were incubated with corresponding secondary antibody (anti-rabbit IgG-HRP, 1:200). Counterstaining was performed using hematoxylin. For the semiquantitative analysis of the immunohistochemical results, three sections from each brain, with each section containing three microscopic fields from the ischemic cerebral cortex, were digitized under a ×200 objective. The immunoreactivity of the target proteins was quantified based on the integrated optical density of immunostaining per field using Image J software.

### Western Blot Assay

The brains were prepared, washed with cold phosphate buffer solution (PBS), resuspended in a lysis buffer, and sonicated the lysate. The proteins were separated on 10% sodium dodecyl sulfate (SDS) gels and transferred to poly-vinylidene fluoride membranes. After incubation with 1:1,000 primary antibody–ACE dilution buffer for 1 h, Goat Anti-Rabbit IgG (H + L) HRP (1:3,000, absin) was used as secondary antibody and developed by enhanced chemiluminescence.

### Molecular Docking Analysis

Molecular docking analysis between *l-*borneol and were performed using Discovery Studio 3.5. The human X-ray crystal structures of VEGF, Ang1, BDNF, Tie2, TGF-β1, MMP9 were obtained from the Protein Data Bank (PDB) archives and used as target for molecular docking. Table 5 showed that the information about those targets PDB ID. The structure of *l-*borneol was drawn by ChemDraw. The active site was searched via “Receptor - Ligand interactions - Define and Edit Bind Site”. The interactions between *l-*borneol and target proteins were detected to obtain the score using LibDock function.

**TABLE 5:** The information of target proteins.

Gene	Name	PDB ID
Ang1	Angiopoietin-1	4epu
BDNF	Brain-derived neurotrophic factor	1b8m
VEGF	Vascular endothelial growth factor	1wq9
TGF-β1	Transforming growth factor-1β	5vqp
Tie2	TEK tyrosine kinase, endothelial	3l8p
MMP9	Matrix metalloproteinase 9	4h82

### Statistical Analysis

Data are expressed as mean ± SD. GraphPad Prism 8 (GraphPad Software Inc., San Diego, CA, United States) was used to analyze all data. Two or multiple groups were compared using Student's unpaired *t*-test or one-way ANOVA followed by Bonferroni *post hoc* test with F at *p* < 0.05 and no significant variance inhomogeneity. *p* < 0.05 was considered statistically significant.

## Results

### Effect of *l-*Borneol Interventions on Neurological Function Score

**FIGURE 2:**
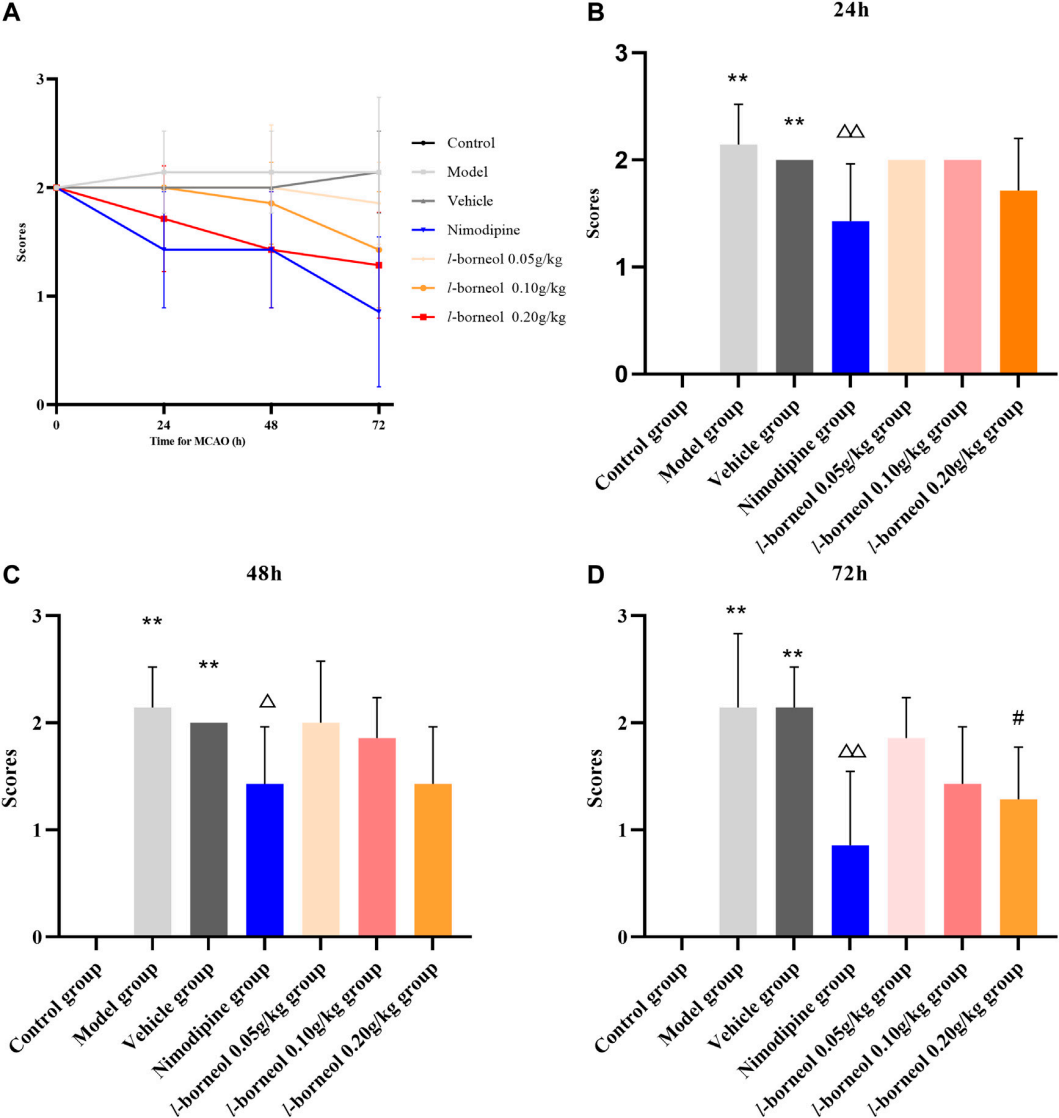
Illustration of neurological function score using Zea–Longa methods (*n* = 6). **(A)** the trend after MCAO; **(B)** neurological function score after 24h; **(C)** neurological function score after 48h; **(D)** neurological function score after 72 h. ^**^ *p* < 0.01, compared with the control group; ^△^ *p* < 0.05, ^△△^ *p* < 0.01, compared with the model group; ^#^ *p* < 0.05, compared with the vehicle group.

Neurological function score was performed by Zea - Longa methods. The results showed in Figure 2. The trend of neurological score after MCAO is shown in Figure 2A, which suggested *l-*borneol post treatment could gradually improve the neurological function. In the day of MCAO, the rats in the control group had no neurological damage, so the score was 0, and the rats in the other groups were scored 2, indicating a successful modeling. The model and vehicle group had no significant difference, it is suggested that 5% Tween solution as a solvent has no significant effect on neurological function after cerebral ischemia. Compared with model group, nimodipine could significantly improve neurological function scores after 24 h, 48 h and 72 h (*p* < 0.05 or *p* < 0.01). Compared with vehicle group, *l-*borneol 0.2 g/kg could significantly reduce the neurological function scores after 72 h (*p* < 0.05). The other time points had the trend of improving the neural function, but there was no significant difference (*p* > 0.05). The results suggest that *l*-borneol can significantly reduce neurological deficits caused by cerebral ischemia.

### Effect of *l-*Borneol Interventions on Cerebral Infarction

**FIGURE 3:**
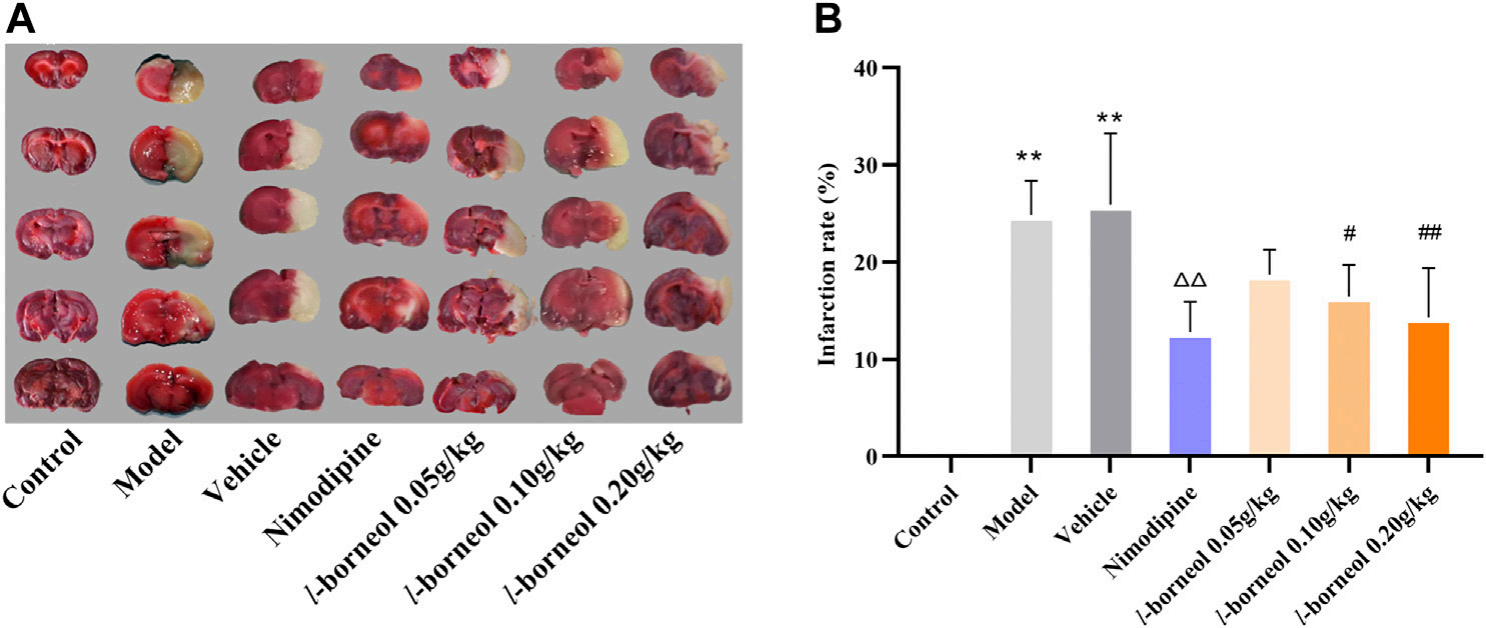
Illustration of cerebral infarction by TTC staining (*n* = 6). The infarcted cells showed white or yellow, the normal side was red. ^**^ *p* < 0.01, compared with the control group; ^△△^ *p* < 0.01, compared with the model group; ^#^ *p* < 0.05, ^##^ *p* < 0.01, compared with the vehicle group.

For the assessment of infarction, TTC staining was used. TTC is converted to red formazone pigment by NAD and dehydrogenase and there of stained the viable cells deep red. The infarcted cells have lost the enzyme and cofactor and thus remained unstained dull yellow or white. The results showed in Figure 3, the rats in the control group had no significant infarct volume, the infarction of the model group and vehicle group showed significantly different from that of the control group (*p* < 0.01). The model and vehicle group had no significant difference. Cerebral infarct size was obviously reduced in the nimodipine group compared with the model group (*p* < 0.01). Compared with the vehicle group, *l-*borneol could reduce the cerebral infarction rate (*p* < 0.05 or *p* < 0.01), showing the dose dependent relationship.

### Effect of *l-*Borneol Interventions on VEGF, Ang1 and Tie two in the Serum

**FIGURE 4:**
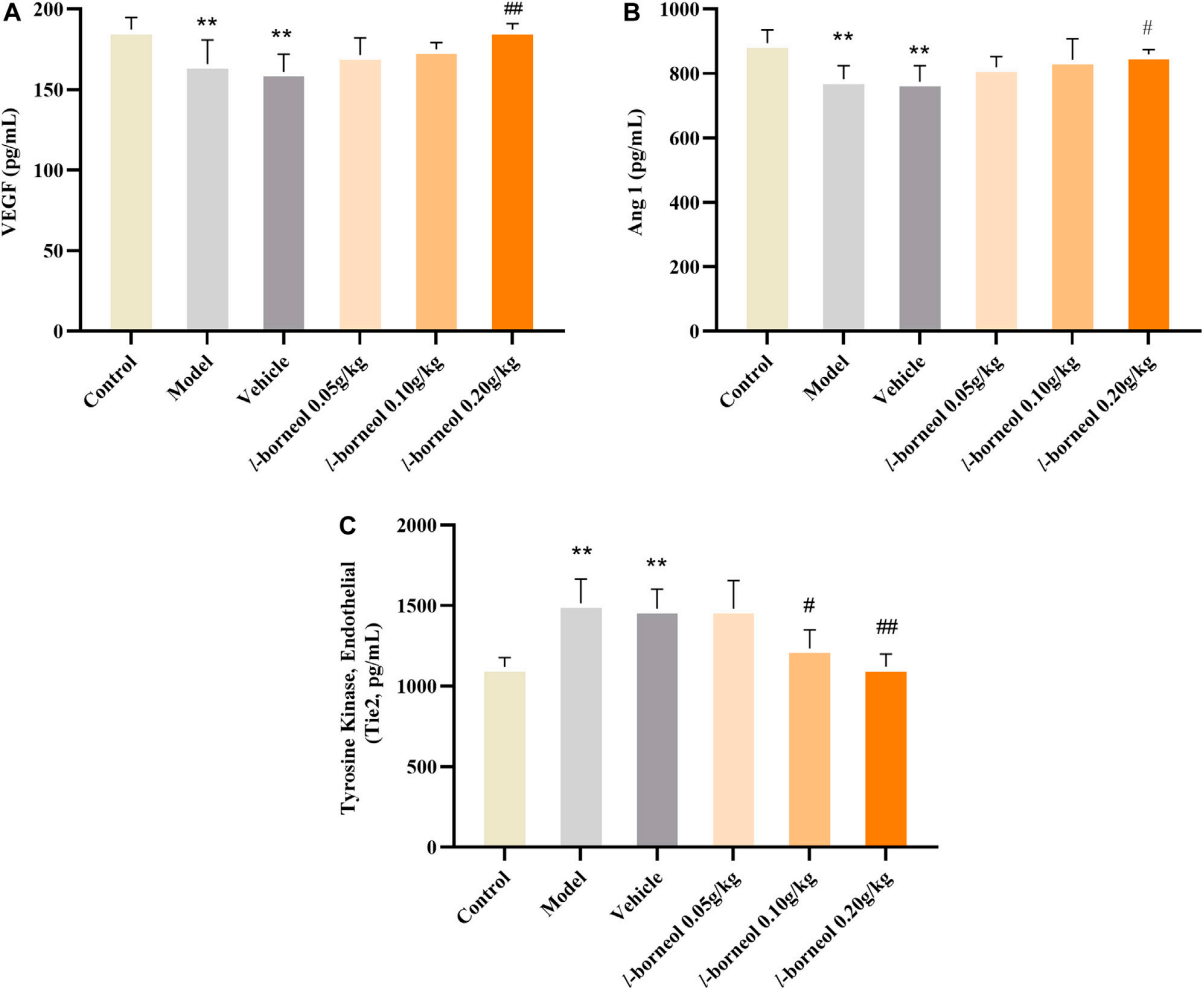
Illustration of VEGF, Ang1 and Tie two in the serum using ELISA (*n* = 6). ^**^ *p* < 0.01, compared with the control group; ^#^ *p* < 0.05, ^##^ *p* < 0.01, compared with the vehicle group.

The levels of VEGF, Ang1 and Tie two in the serum were performed by ELISA methods. The results showed in Figure 4. The expressions of angiogenic growth factors including VEGF and Ang-1 in the model and vehicle group were significantly decreased, while the expression of Tie two was significantly increased as compared with those in the control group (*p* < 0.01). The model and vehicle group had no significant difference. Compared with the vehicle group, VEGF and Ang-1 expression levels significantly increased in the *l-*borneol 0.2 g/kg groups (*p* < 0.01 or *p* < 0.05), Tie two expression in the *l-*borneol 0.1 and 0.2 g/kg group was significantly decreased (*p* < 0.05 or *p* < 0.01). These results demonstrate that *l-*borneol could promote angiogenesis in a dose-dependent manner against ischemic brain injury.

### Effect of *l-*Borneol Interventions on TGF-β1, BDNF, MMP9 in the Serum

**FIGURE 5:**
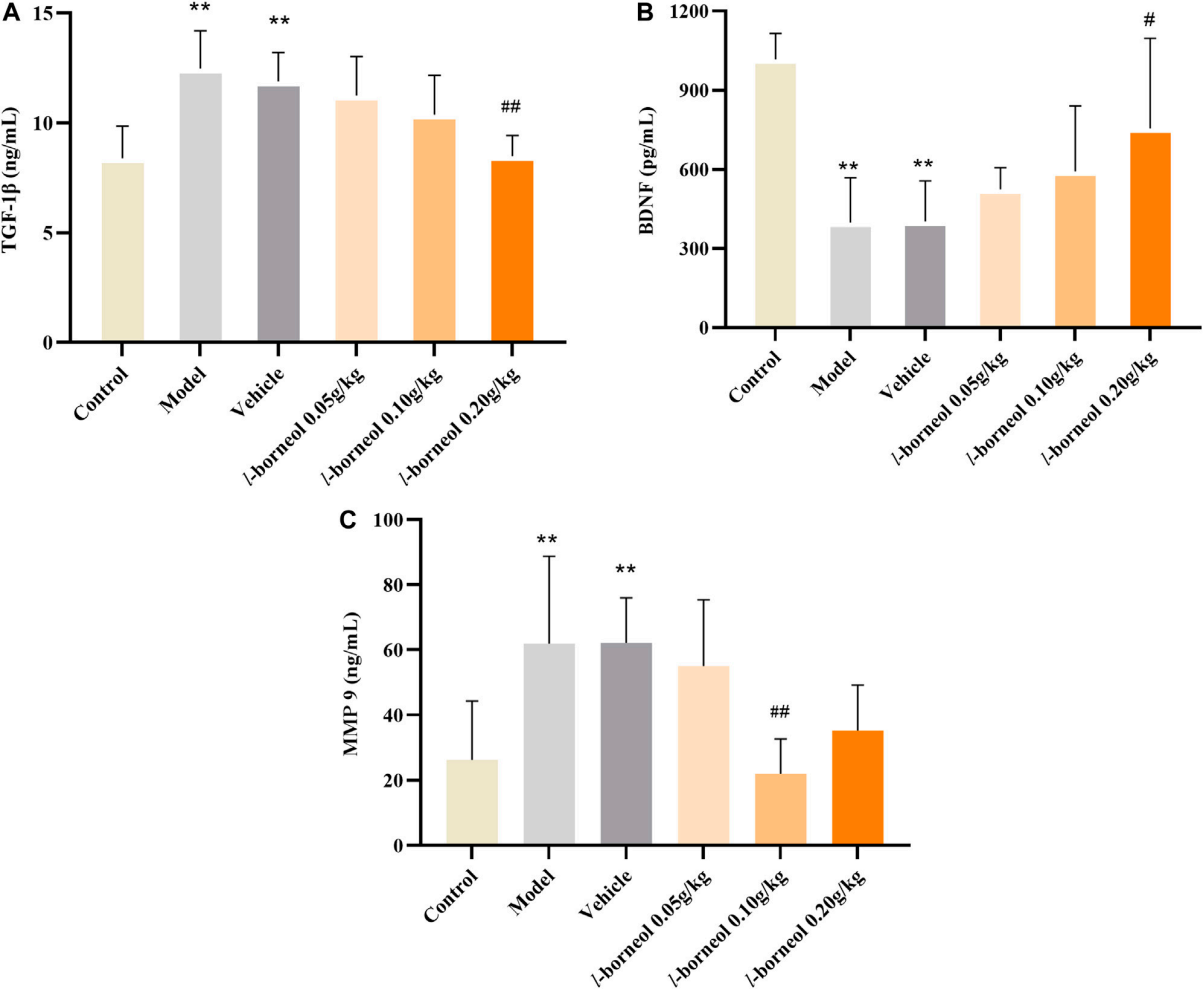
Illustration of TGF-β1, BDNF, MMP9 in the serum using ELISA (*n* = 6). ^**^ *p* < 0.01, compared with the control group; ^#^ *p* < 0.05, ^##^ *p* < 0.01, compared with the vehicle group.

The levels of TGF-β1, BDNF, MMP9 in the serum were performed by ELISA methods. The results showed in Figure 5. Compared with control group, the expressions TGF-β1 and MMP9 were significantly increased in the model and vehicle group, the expressions level of BDNF was significantly decreased in the model and vehicle group (*p* < 0.01). The model and vehicle group had no significant difference. While, the expressions of TGF-β1 in the *l-*borneol 0.2 g/kg group was significantly decreased (*p* < 0.01), and the BDNF was significantly increased as compared with those in the vehicle control group (*p* < 0.05), and presenting dosing dependent. The expressions level of MMP9 was significantly decreased in *l-*borneol 0.1 g/kg group (*p* < 0.01). It suggested *l-*borneol could promote neurogenesis against ischemic brain injury.

### Effect of *l-*Borneol Interventions on Pathological Changes in Brain

**FIGURE 6:**
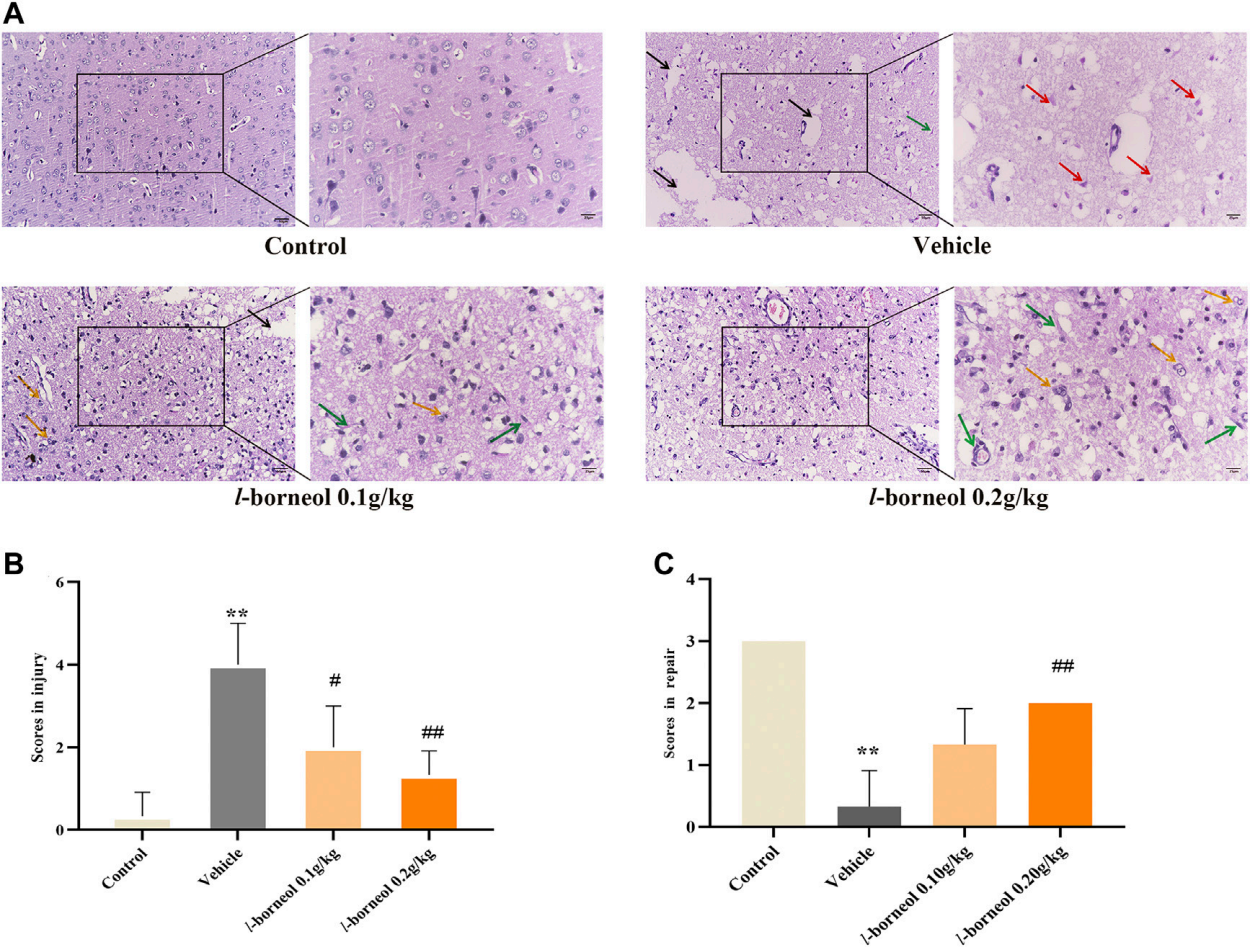
Illustration of pathological changes using HE staining (*n* = 3). **(A)** HE results of each group, **(B)** Semi-quantitative analysis of pathological injury, **(C)** Semi-quantitative analysis of pathological repair. Red arrow represents red neuron, black arrow represents liquefied and necrotic nerve tissue, yellow arrow represents microglia clears out necrotic neurons, green arrows represents new blood vessels, ^**^ *p* < 0.01, compared with the control group; ^#^ *p* < 0.05, ^##^ *p* < 0.01, compared with the vehicle group.

HE staining was performed to obverse the pathological changes under light microscope with ×200 and ×400 magnification. The morphology and structure of the cerebral cortex were normal without obvious pathological changes in the control group. And there were many blood vessels full of red blood cells in some fields (Figure 6A). There was obvious infarct, the homogenized cells, liquefied and necrotic nerve tissue to form a cribriform focus in the vehicle group. And shows a large number of red neurons, which is a sign of acute ischemic injury. What’s more, the cell density was obviously reduced and there was swelling phenomenon, which is different from control group. Infarct size were found in *l-*borneol 0.1 and 0.2 g/kg, while the degree of ischemic damage was relatively light (Figure 6A). It was found that there was the decrease of liquefied necrosis area, rare red neurons, more capillaries, higher cell density and obvious phagocytosis of proliferative glial cells than the vehicle group. According to the results of semi-quantitative scoring of tissue injury and tissue repair (Figures 6B,C), the brain injury was significant reduced in *l-*borneol 0.1 and 0.2 g/kg group (*p* < 0.01, *p* < 0.05), *l-*borneol 0.2 g/kg group had more brain repair effect than vehicle group (*p* < 0.01), presenting vessels production and glial cells clear out necrotic neurons.

### Effect of *l-*Borneol Interventions on ACE, CD34, HIF 1α mRNA in Brain

**FIGURE 7:**
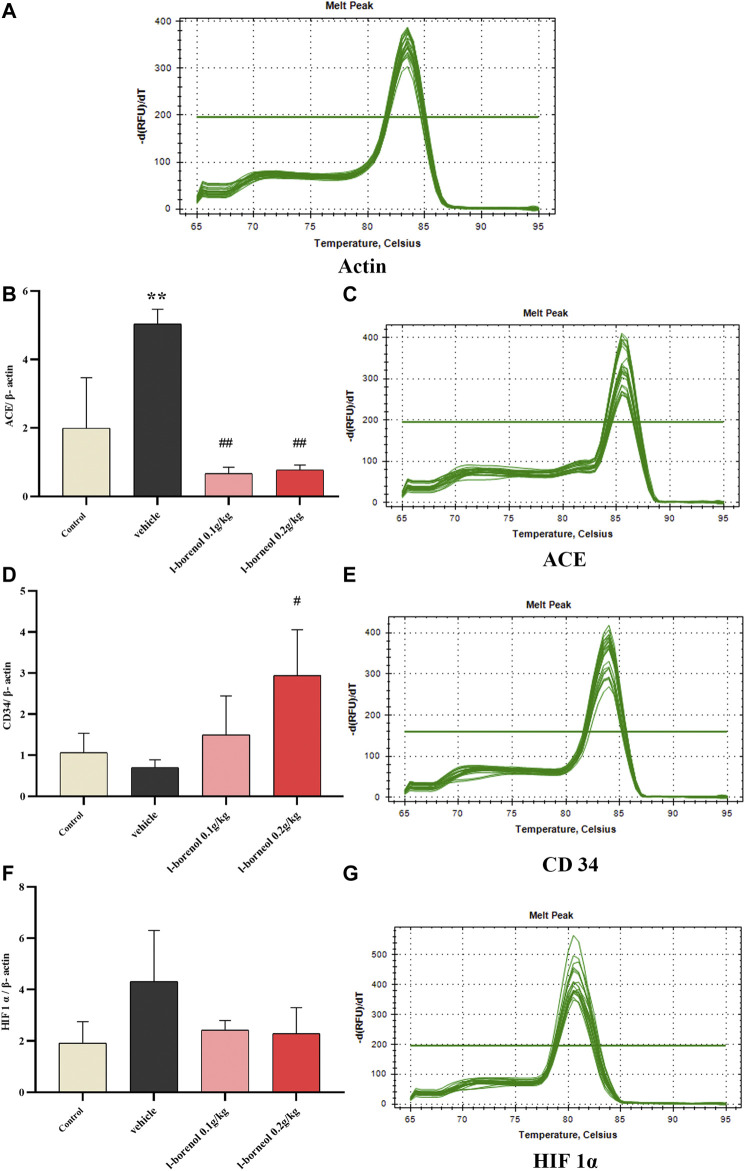
Illustration of ACE, CD34, HIF 1α mRNA using qRT-PCR (*n* = 3). ^**^ *p* < 0.01, compared with the control group; ^#^ *p* < 0.05, ^##^ *p* < 0.01, compared with the vehicle group.

The ACE, CD34, HIF 1α mRNA expression was detected by qRT-PCR (Figure 7). Compared with control group, the expression of ACE mRNA was obviously increased in the vehicle group (*p* < 0.01), the expression of HIF1αmRNA showed an increasing trend, and the expression of CD34 mRNA showed a decreasing trend. Compared with vehicle group, *l-*borneol 0.1 and 0.2 g/kg could significantly reduce ACE expression (*p* < 0.01) and *l-*borneol 0.2 g/kg also increased CD 34 expression (*p* < 0.05). The expression of HIF1α mRNA in the *l*-borneol 0.1 and 0.2 g/kg groups showed a downward trend.

### Effect of *l-*Borneol Interventions on ACE in Brain

**FIGURE 8:**
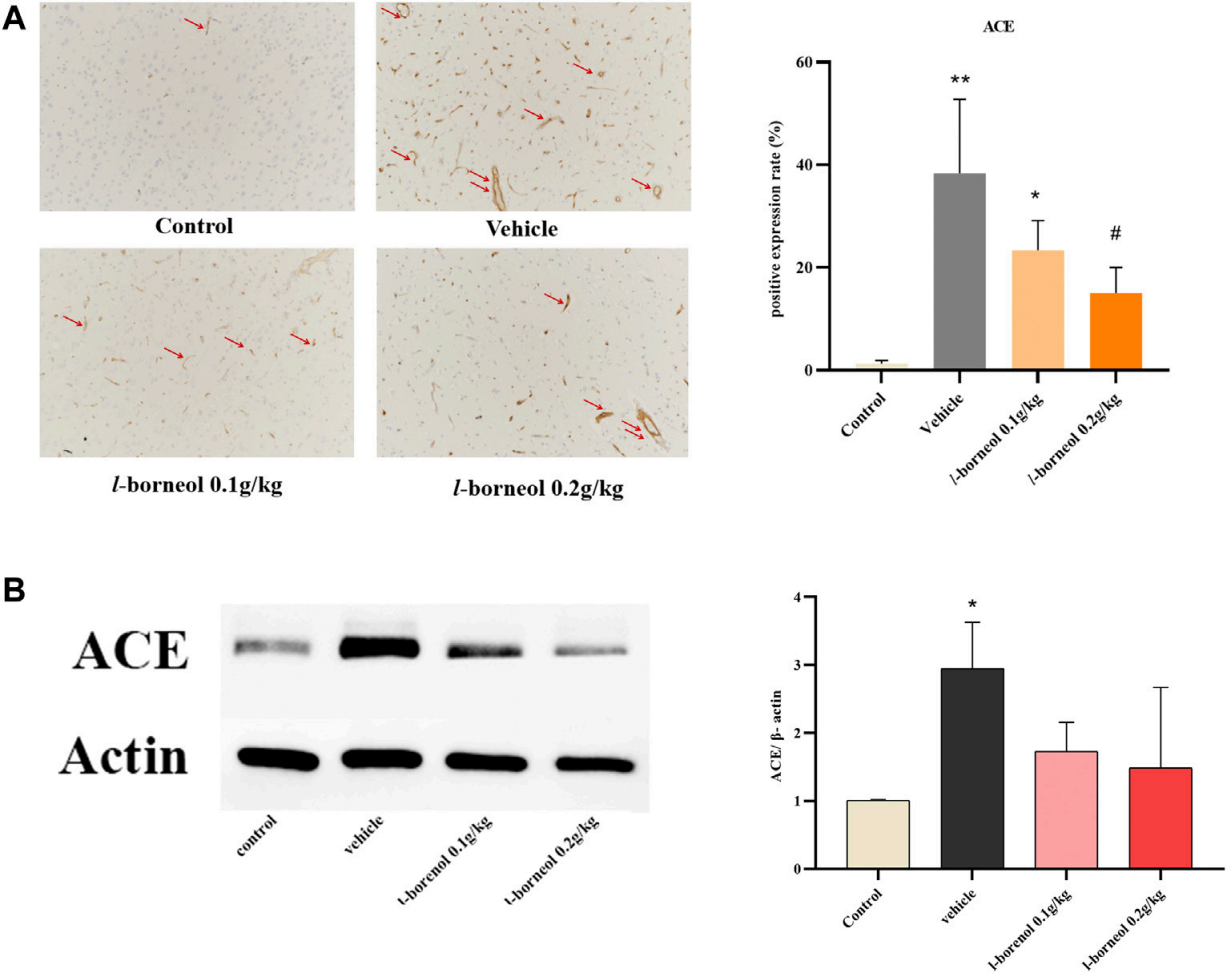
Illustration of ACE expression using IHC staining and western blot (*n* = 3). ^**^ *p* < 0.01, compared with the control group; ^#^ *p* < 0.05, ^##^ *p* < 0.01, compared with the vehicle group, red arrow indicates positive expression.

The effect of *l-*borneol interventions on blood vessels was measured by IHC staining using ACE antibody to mark vessels. The results showed that the number of ACE -positive cells was markedly elevated after MCAO than those of control group, However, the increased number of ACE -positive cells were attenuated by *l-*borneol (Figure 8A). The results of western blot also showed, *l-*borneol had a trend of reducing ACE expression compared with vehicle group (Figure 8B).

### Effect of *l-*Borneol Interventions on VEGFR and CD 34 in Brain

**FIGURE 9:**
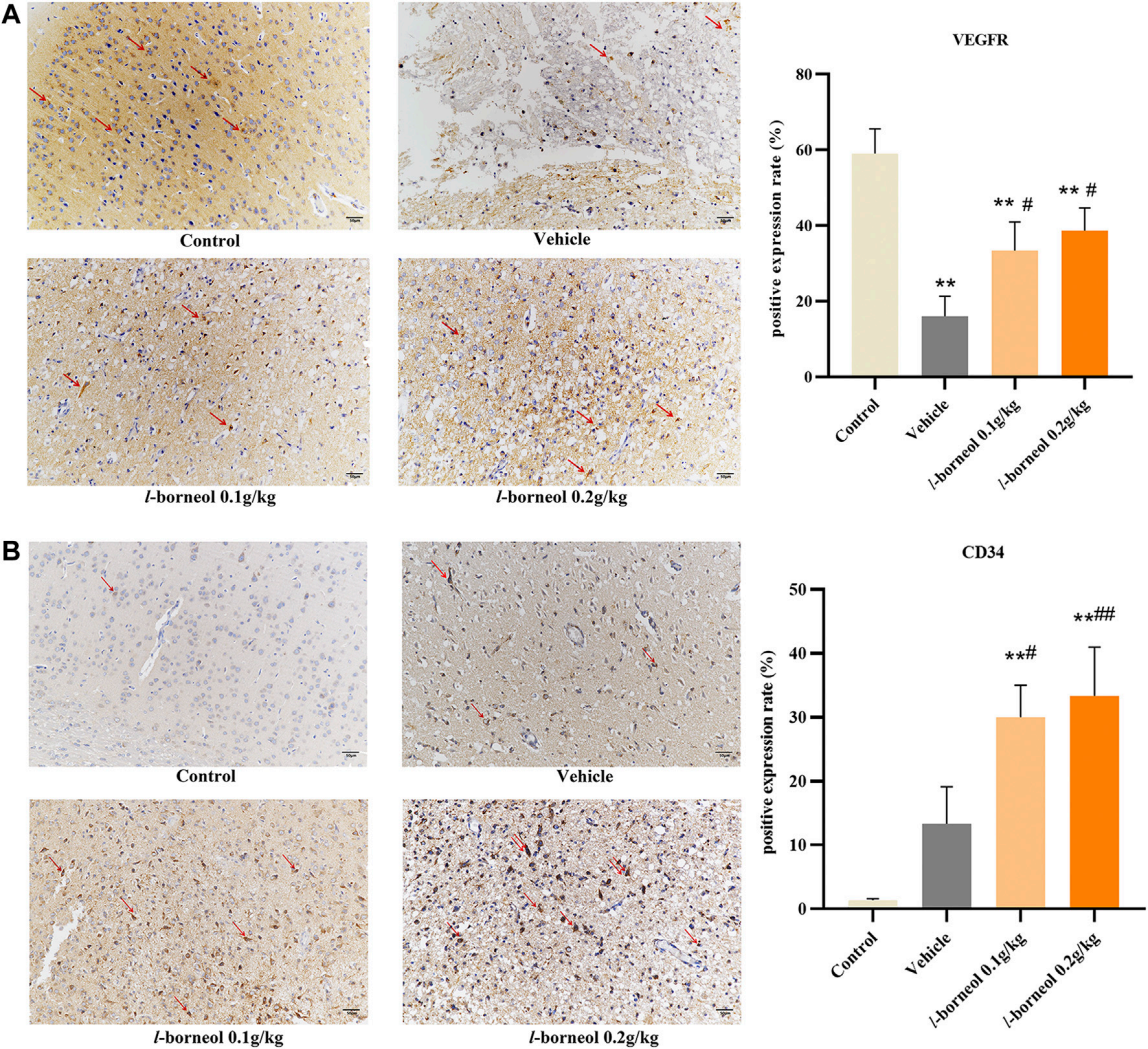
Illustration of VEGFR and CD34 expression using IHC staining (*n* = 3). ^**^ *p* < 0.01, compared with the control group; ^#^ *p* < 0.05, ^##^ *p* < 0.01, compared with the vehicle group, red arrow indicates positive expression.

The effect of *l-*borneol interventions on vascular endothelial growth factor receptor (VEGFR) was measured by IHC staining using VEGFR antibody. The results showed that the number of VEGFR -positive cells was markedly reduced after MCAO than those of control group, However, the number of VEGFR -positive cells were increased by *l-*borneol (Figure 9A). In addition, we use CD34 antibody to mark blood vessels by IHC staining. CD34 staining was performed as an indicator of microvessel density (MVD). The results showed the number of CD34-positive cells was increased after MCAO than that of control group. This suggests that ischemia activates the endogenous angiogenesis compensatory improvement of ischemic damage. The number of CD34-positive cells was still markedly increased after MCAO with *l-*borneol interventions than that of control group (Figure 9B), suggesting *l-*borneol increased the number of the MVD, further promotes angiogenesis.

### The Overall Effect of L-Borneol Intervention on Cerebral Ischemia Related Factors

**FIGURE 10:**
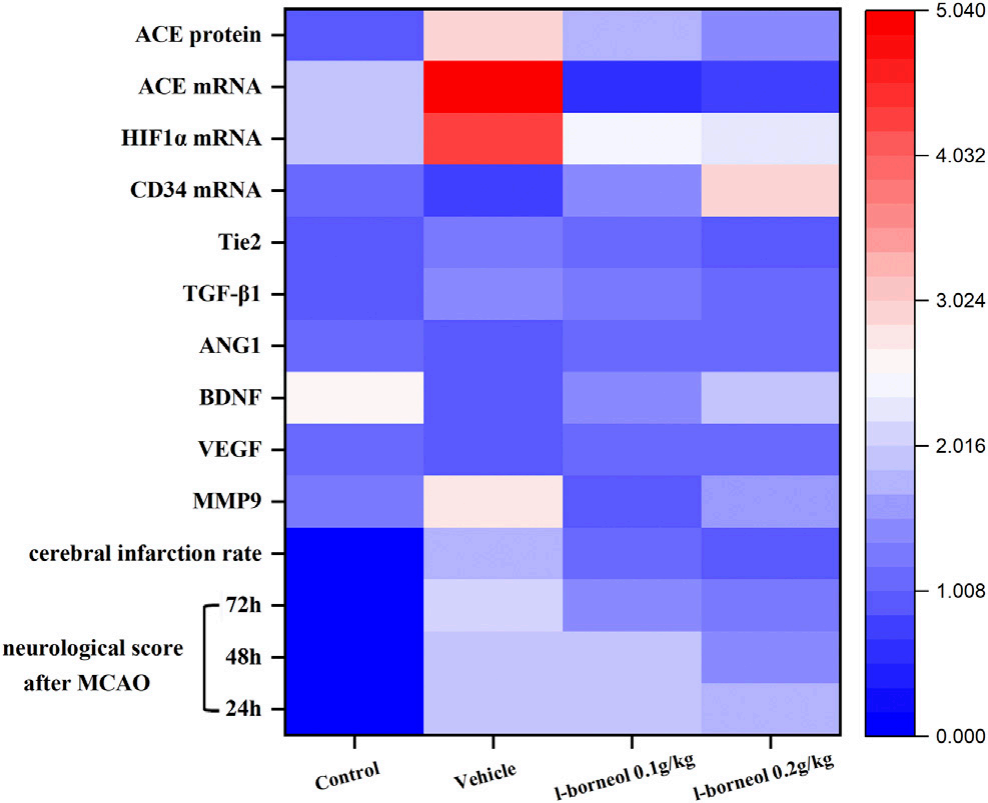
Heat map results of *l-*borneol on various indicators.

The heat map can visualize the data, and integrate the indicators to intuitively compare the strength of their expression. The heat map shows the overall regulation of *l-*borneol on cerebral ischemia and mechanism factors (Figure 10). *l-*borneol promotes angiogenesis and neurogenesis by increasing the levels of Ang 1, VEGF and BDNF after cerebral ischemia. The increase of CD34 protein confirms this result. *l-*borneol can also inhibit neuronal apoptosis and enhance the stability of neovascularization by inhibiting the levels of ACE, MMP9, HIF1α, TGF-β1 and Tie2, thereby improving the neurological deficit in ischemic rats, reducing the rate of cerebral infarction, and exerting neuroprotective effects.

### Molecular Docking Analysis

**FIGURE 11:**
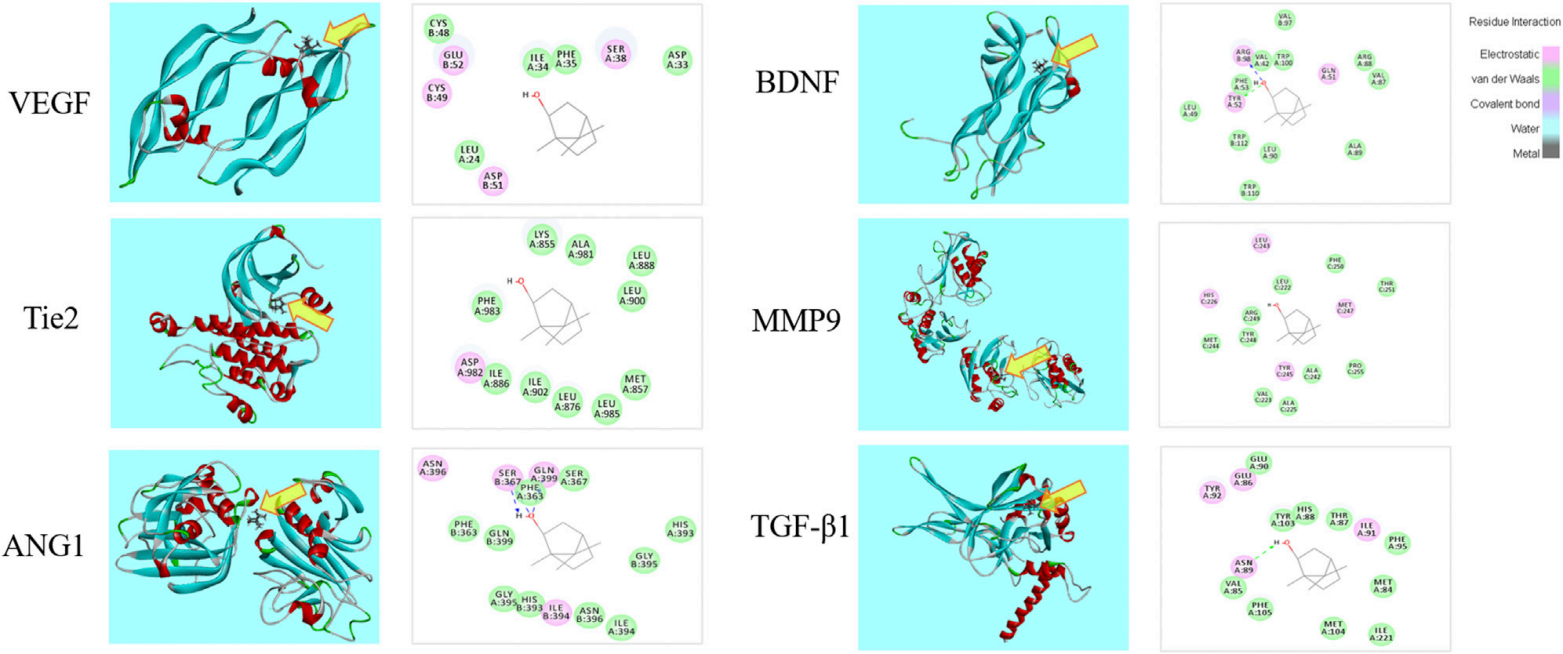
Graph of the small molecule and the target protein.

The 3D and 2D binding graphs of the small molecule and the target protein after docking are shown in Figure 11, and the binding scores are shown in Table 6. The results showed that *l-*borneol has certain binding effect with VEGF, Tie2, Ang1, TGF - β1, BDNF, MMP9, mainly through hydrogen bonding.

**TABLE 6:** The scores of the small molecule and the target protein.

Gene	PDB ID	Score	Gene	PDB ID	Score
VEGF	1wq9	40.12	BDNF	1b8m	66.68
Tie2	3l8p	45.14	MMP9	4h82	63.46
ANG1	4epu	69.06	TGF-β1	5vqp	72.49

## Discussion

Stroke is a disease caused by insufficient blood supply and vascular embolism, which has the characteristics of high incidence rate, high mortality rate, high recurrence rate and high disability rate. In 2015, according to the World Health Organization, stroke which is the second leading cause of death caused 6.2 million deaths globally. With the aging of the population, the incidence rate is increasing year by year. Moreover, with the rapid development of modern life and the gradual increase of modern pressure, the incidence of stroke tends to encroach on young people and bring a heavy burden to society and families (Boot et al., 2020). At present, there is no ideal treatment strategy. Therefore, the development of new drugs and new treatment strategies is still an urgent problem in the treatment of stroke. Borneol is a traditional Chinese medicine, which has the effect of resuscitation and restoring the spirit. The Chinese patent medicine containing borneol is commonly used in the treatment of cerebrovascular diseases, and even has better therapeutic effect than other therapies (Liu et al., 2019; Ma et al., 2018 and 2017). *L-*borneol comes from *Blumea balsamiera (L.)* DC, which is safe and easy to obtain (Luo et al., 2018). Its curative effect is equivalent to that of natural borneol. The previous study showed that the order of the efficacy against cerebral ischemia injury was *l-*borneol, natural borneol, synthetic borneol (Dong et al., 2018). Therefore, this study is to investigate the angiogenesis and neurogenesis mechanism of *l-*borneol against cerebral ischemia.

In the current study, we explored the neuroprotection of *l-*borneol, a natural small molecule, against cerebral ischemia injury and endeavored to elucidate its underlying mechanisms. Our findings demonstrated that *l-*borneol effectively protected against outcomes of cerebral ischemia injury as evidenced by reduced MCAO-induced neurological deficits, cerebral infarction size. Therefore, it is reasonable to believe that *l-*borneol might be certain brain protective effect against cerebral ischemia injury. Hypertension is a high risk factor for stroke. ACE is an important metabolic enzyme in the RAS system that regulates blood pressure. It can convert inactive Ang I into Ang II, which in turn acts on the AT1R, inducing vasoconstriction, increasing blood pressure and aggravates ischemic damage. It's reported that ACE was significantly increased in the acute stage of cerebral ischemic stroke (Lu et al., 2013), thus reducing ACE levels can alleviate ischemic injury. Regarding clinical studies, treatment with ACE inhibitors and AT1 receptor antagonists exert preventive and therapeutic effects on stroke (Kangussu et al., 2019). In addition, Ang II can induce the expression and activation of MMP9 via NF-KappaB dependent pathway. In turn, it may lead to instability and rupture of atherosclerotic plaque or cerebral aneurysm, thereby triggering stroke. It also degrades the extracellular matrix after stroke, promotes BBB leakage, causes vasogenic edema, and destroys the stability of new blood vessels. Therefore, reducing the level of MMP9 helps to reduce the permeability of new blood vessels and strengthen the stability of the vessel wall (Turner and Sharp, 2016). In our study, the results showed that *l*-borneol can significantly inhibit the activity of ACE and reduce the level of MMP9, suggesting that *l*-borneol can reduce ischemic damage and strengthen the stability of new blood vessels.

As noted, angiogenesis and neurogenesis play important roles in neuroprotection (Seevinck et al., 2010; Benedek et al., 2019; Zhang et al., 2020). HIF-1α can be activated after ischemia, which is the earliest cytokine involved in the specific response of cells to hypoxia. It can regulate the expression of various factors such as Ang and VEGF to promote angiogenesis, so that the body can better adapt to hypoxia environment. However, in the early stage of ischemia, overexpression of HIF-1α can induce cell apoptosis. Studies have shown that HIF1α knockout mice show better survival rates and improved neurological function in the early stage of ischemia. Therefore, the early inhibition of HIF1a has a certain anti-apoptotic effect and reduces nerve function damage (Barteczek et al., 2017). HIF-1α can induce the release of Ang1, VEGF and BDNF from vascular endothelial cells, and promote angiogenesis and neurogenesis. However, Ang1 and VEGF have different effects in inducing angiogenesis. VEGF is a powerful endothelial cell mitogen, which can promote the proliferation and aggregation of vascular endothelial cells to form a lumen. However, VEGF is also known as vascular permeability factor. The neovascularization induced by VEGF is usually immature and leaking, which may cause brain edema and aggravate brain damage (Couvelard et al., 2000; Lena, 2016; Jin et al., 2017) Therefore, it needs to cooperate with Ang one to induce angiogenesis. Tie two is the specific receptor of Ang1 (Dixit et al., 2007; Saharinen et al., 2017), Ang-1 promotes endothelial cell budding, migration and chemotaxis by activating Tie 2 (Saharinen et al., 2017), and reduce the vascular permeability caused by VEGF, MMP9 and other factors. It can also maintain the stability of the vascular structure by promoting the interaction between cells and cells, cells and substrates (Shen et al., 2011). VEGF and Ang one promote angiogenesis and maintain vascular stability through coordinate with each other (Zhang and Chopp, 2002).

CD34 antigen is a stage-specific leukocyte differentiation antigen, which is selectively expressed on the surface of humanstemcell, progenitorcell and vascular endothelial cells. Because its expression in neovascular endothelium is much greater than that in non-neovascular endothelium, it becomes the most sensitive vascular endothelial marker. It is often used to count the density of neovascularization to determine angiogenesis. (Solmaz and Guvena, 2016). In our study, *l*-borneol can effectively increase the levels of VEGF in serum and VEGF mRNA in tissues, and increase the expression of VEGFR in the cortex of brain tissue, while regulating the Ang1/Tie2 pathway, synergistically VEGF promotes angiogenesis and stabilizes the structure of blood vessels. CD34mRNA expression and CD34 positive cells in brain tissue increased significantly after *l*-borneol administration, indicating the increasing number of MVD, which further confirmed the angiogenesis effect of *l*-borneol. In addition, *l*-borneol can significantly inhibit cell apoptosis induced by HIF-1α transcription, and can significantly inhibit the levels of ACE and MMP9 in brain tissue to further alleviate cerebral ischemic damage.

Cerebral ischemia induced the death of neurons is the main cause of neuron dysfunction. Therefore, increasing neurons survival and regeneration in cerebral penumbra is an important means to improve the neuron function (Puig et al., 2018). TGF-β1 is an important regulator in the transformation and growth of cells and angiogenesis (Lupo et al., 2014). It could stimulate neuron to secrete cell growth factors, such as VEGF, BDNF, and promote the survival and differentiation of neurons (Guan and Sun, 2002).

Studies have demonstrated that BDNF can promote the proliferation, differentiation and migration of neural stem cells (NSC), promote the growth of axons and dendrites in the process of neuron growth, and participate in the formation and maturation of synapses. As a principle of mitosis, VEGF can directly promote the proliferation of neural precursor cells, and stimulate the proliferation and differentiation of neural precursor cells by promoting the establishment of vascular niches. In addition, VEGF can further induce neurogenesis by stimulating endothelial cells to release BDNF. Exogenously induced VEGF overexpression can increase the conversion rate of neurons, enhance neurogenesis and neural migration rate (Wang et al., 2007a; Wang et al., 2007b). VEGF and BDNF can mediate the coupling loop of neurogenesis and angiogenesis. NSC induces endothelial cells to release VEGF and BDNF by promoting the up-regulation of nitric oxide (NO). VEGF and BDNF activate VEGFR and TrkB on endothelial cells to induce angiogenesis through autocrine and paracrine methods. VEGF and BDNF can also promote the phosphorylation of eNOS (endothelial nitric oxide synthases) on NSC, continue to produce NO and stimulate the proliferation of NSC, and promote neurogenesis, thereby forming a feedback loop of angiogenesis coupled neurogenesis with VEGF and BDNF as targets.

In our study, *l*-borneol significantly increased the levels of VEGF and BDNF, and increased the number of VEGFR in the cortex of brain tissue, suggesting that *l*-borneol can promote neurogenesis by increasing the expression of VEGF and BDNF. In addition, the results show that *l*-borneol can significantly reduce the level of TGF-β1. Studies have shown that TGF-β1 has a two-way effect, and low concentrations of TGF-β1 have a positive synergistic effect on angiogenesis. The high concentration of TGF-β1 can reduce the VEGF-mediated pro-angiogenesis effect (Dobolyi et al., 2012), and has the effect of inducing MMP9 activity. *l*-borneol may reduce the concentration of TGF-β1, enhance angiogenesis, reduce the level of MMP9, and stabilize vascular permeability. In conclusion, our research shows that *l*-borneol can improve ischemic injury by inhibiting the expression of HIF-1α, ACE, TGF-β1 and MMP9, and regulate the Ang1-VEGF-BDNF pathway to promote angiogenesis coupled neurogenesis to exert neuroprotective effects. Molecular docking also confirmed that the above factors may be the targets of *l*-borneol to exert neuroprotective effects.

## Conclusion

**FIGURE 12:**
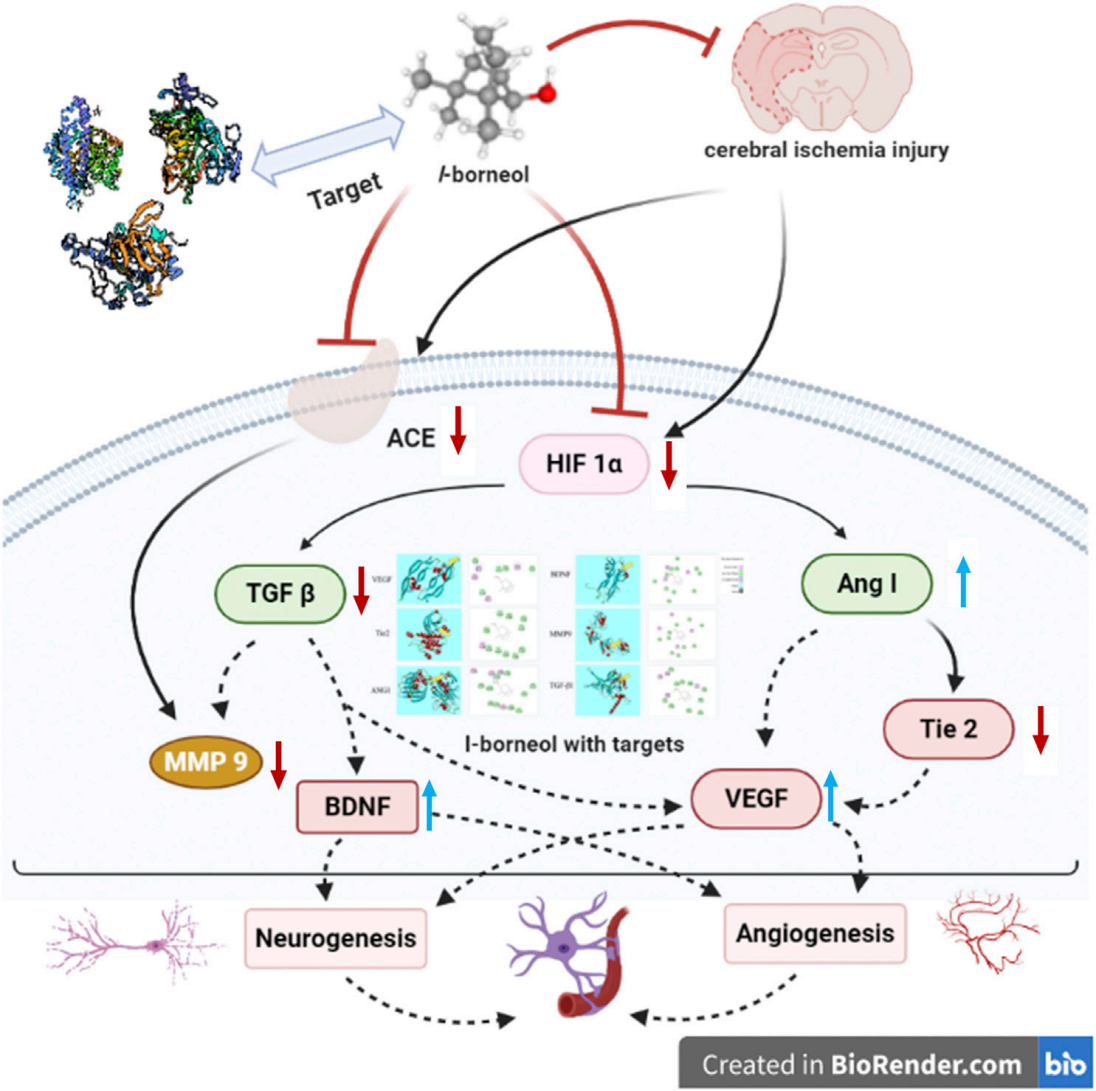
Schematic presentation of a proposed mechanism for the protective role of *l*-borneol against cerebral ischemia injury (https://app.biorender.com/). Red arrows represent an inhibition effect; Blue arrows represent an enhancement effect.

Our study indicated that *l*-borneol had a significant effect on improving neurological function scores and infarction size. Simultaneously, *l*-borneol alleviated the pathological changes in the brain tissue. The neuroprotective effect of *l*-borneol against cerebral ischemia might be associated with angiogenesis and neurogenesis. *l*-borneol could promote Ang-1, VEGF, BDNF expression and enhance micro vessels density, inhibit TGF-β1, Tie 2, MMP9 and ACE, which is the biological basis of the neuroprotective effect of *l*-borneol (Figure 12). Certainly, the mechanisms of injury are complicated, and the pathway on angiogenesis and neurogenesis is various, other mechanisms remains to be evaluated in future studies. In addition, the long-term therapeutic effect of *l*-borneol also needs to be verified in future studies.

## Data Availability

The datasets presented in this study can be found in online repositories. The names of the repository/repositories and accession number(s) can be found in the article/Supplementary Material.

## Abbreviations

t-PA, Tissue plasminogen activator; FDA, Food and Drug Administration; VEGF, vascular endothelial growth factor; Ang, angiopoietin; BDNF, Brain derived neurotrophic factor; SVZ, subventricular zone; LPS, lipopolysaccharide; BBB, blood brain-barrier; MCAO, middle cerebral artery occlusion; SD, Sprague Dawley; TTC, 2,3,5-Triphenyltetrazolium chloride; ELISA, Enzyme-linked immunosorbent assay; Hematoxylin-eosin, HE; IHC, immunohistochemical; ACE, angiotensin-converting enzyme; PBS, Phosphate buffer solution; SDS, Sodium dodecyl sulfate; PDB, Protein Data Bank; TGF-β1, Transforming growth factor, MMP9, Matrix metalloprotein 9; qRT-PCR, quantitative real-time polymerase chain reaction; VEGFR, vascular endothelial growth factor receptor; MVD, Microvessel density; eNOS, endothelial nitric oxide synthases, NO, nitric oxide
