# Supplementary figures and images for: l-Borneol Exerted the Neuroprotective Effect by Promoting Angiogenesis Coupled With Neurogenesis via Ang1-VEGF-BDNF Pathway
Source: Front Pharmacol. 2021 Mar 5;12:641894. doi: 10.3389/fphar.2021.641894 (PMC7973462; doi:10.3389/fphar.2021.641894)

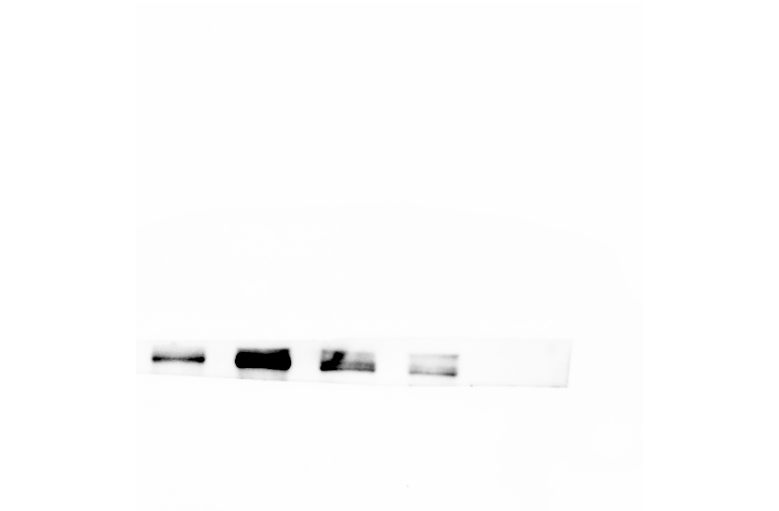

Supplement: Supplementary file 1 [file image1.tif]

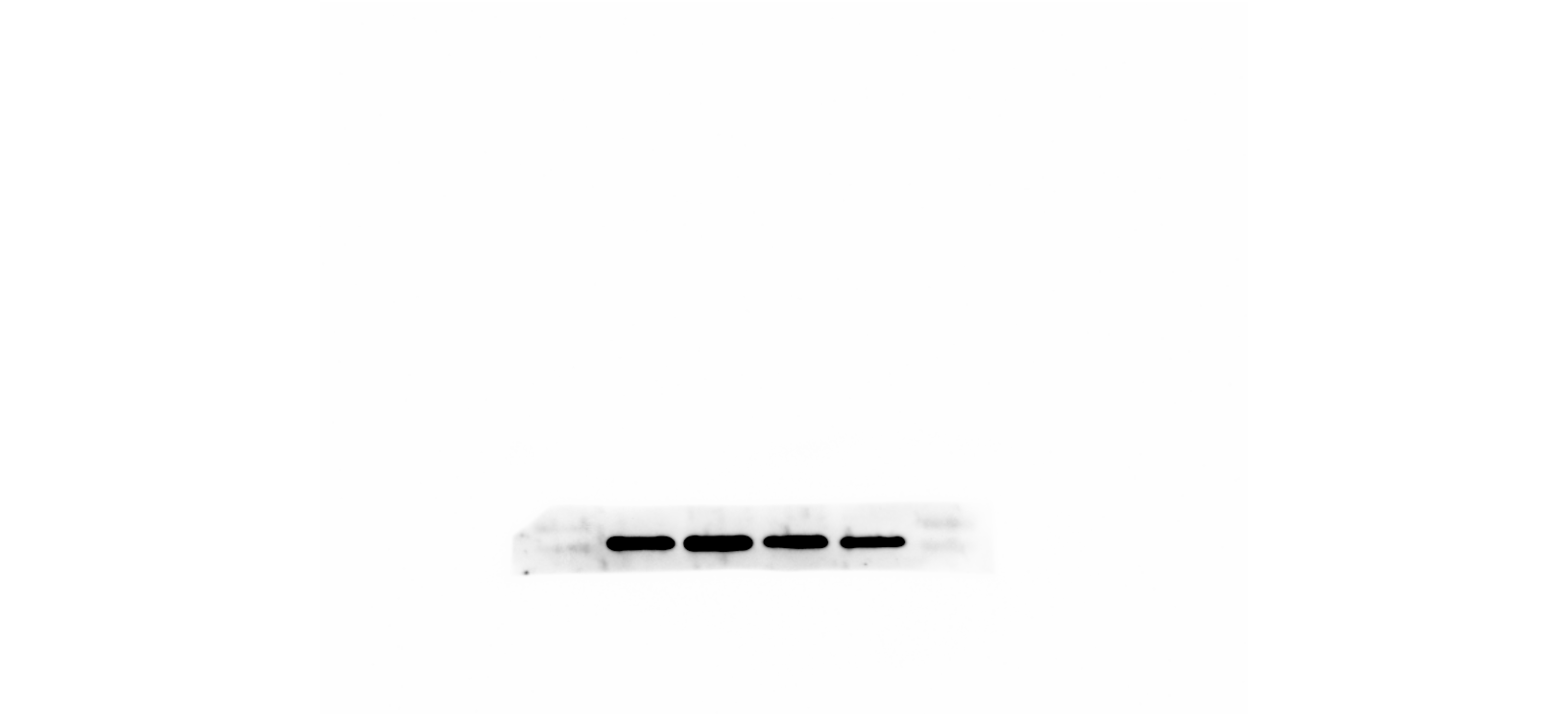

Supplement: Supplementary file 2 [file image2.tif]

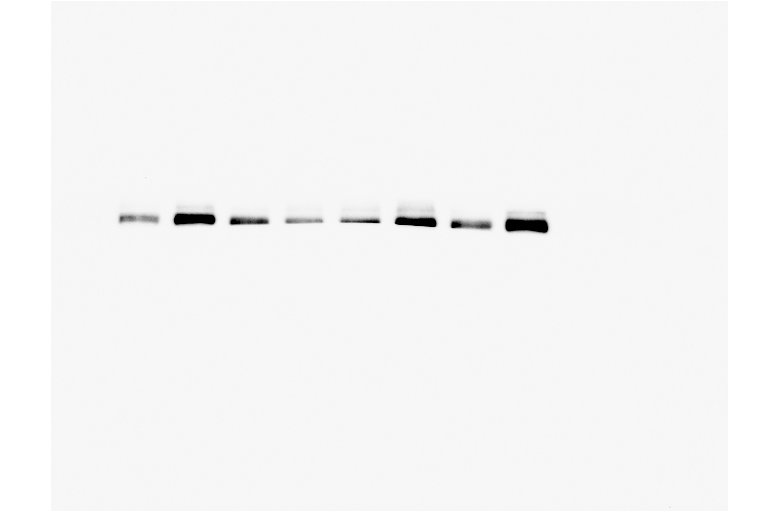

Supplement: Supplementary file 3 [file image3.tif]

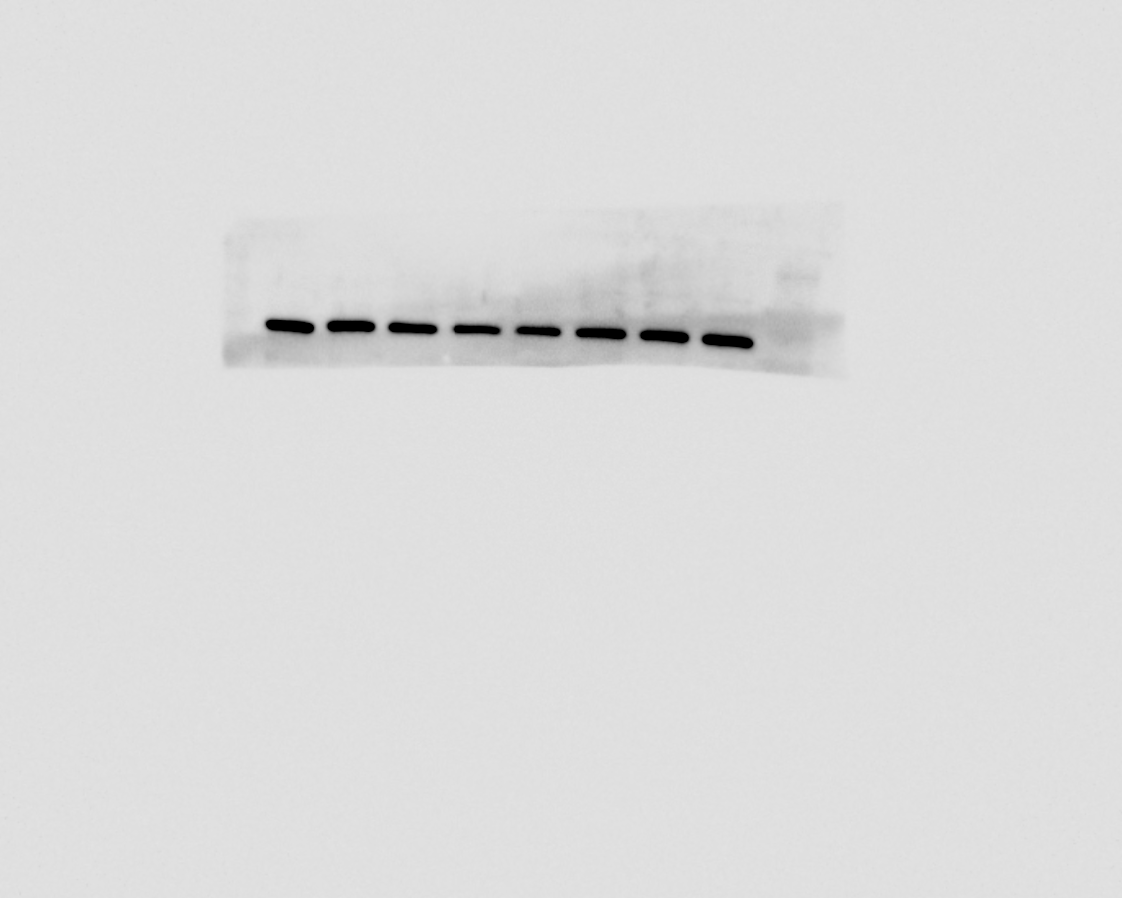

Supplement: Supplementary file 4 [file image4.tif]
